# Survival analysis of time to amputation following diabetic foot ulcer diagnosis among Indonesians

**DOI:** 10.1371/journal.pone.0343399

**Published:** 2026-06-16

**Authors:** Lianawati, Wongsa Laohasiriwong, Kyaw Min Htike, Kavin Thinkhamrop, Roshan Kumar Mahato

**Affiliations:** 1 Doctor of Public Health Program, Faculty of Public Health, Khon Kaen University, Khon Kaen, Thailand; 2 Department of Health Management Innovative Technology, Faculty of Public Health, Khon Kaen University, Khon Kaen, Thailand; University of Iowa Hospitals and Clinics Pathology: The University of Iowa Hospitals and Clinics Department of Pathology, UNITED STATES OF AMERICA

## Abstract

**Background:**

Diabetic foot ulcer (DFU) is a severe complication of diabetes that often leads to amputation and high mortality, yet survival outcomes and risk factors after DFU-related amputation remain poorly understood particularly in Indonesia. This study aims to determine the incidence rate (IR), survival rate and to identify factors associated with the survival of amputation among patients having DFU in Indonesia.

**Methods:**

This retrospective cohort study included Type 2 Diabetes Mellitus (T2DM) patients with DFU admitted to the Margono Hospital in Central Java Indonesia. The primary outcome of this study was the times since diagnosis of DFU to amputation. This study outcome was conducted for 5-year survival (2019–2024), IR and survival rate of amputation. Multiple cox regression was performed to investigate factors associated with survival, quantified by adjusted hazard ratios (AHR) and their 95% CIs.

**Results:**

This cohort comprised 878 DFU patients and followed up for 1,350.55 person-year within the study period. The overall amputation IR was 14.22 per 100 person-year (95% CI: 12.34–16.38) with the highest IR/100 population was 85.68 (95% CI: 73.49–99.90) occurred in the gangrene group. The overall survival rate within 1-year, 3-year and 5-year was 76.14% (95%CI: 72.82–79.11), 72.77% (95%CI: 69.08–76.10) and 71.18% (95% CI: 67.22–74.75) respectively with the highest five-year survival rates were observed among patients with cardiovascular disease (94.37%, 95% CI: 85.17–97.93). Factors that were associated with survival included patients who receiving insulin therapy (AHR: 5.51; 95% CI: 3.73–8.14), residing in rural areas (AHR: 4.14; 95% CI: 1.70–10.12), patients without neuropathy (AHR: 2.40; 95% CI: 1.30–4.42), those with underweight or normal BMI (AHR: 2.04; 95% CI: 1.40–2.98), and those without hypertension (AHR: 1.52; 95% CI: 1.03–2.22).

**Conclusions:**

The study revealed that the rate of amputations was relatively high and highest survival rate was patients with cardiovascular disease. Receiving insulin therapy, staying in rural area, no-neuropathy, underweight and normal weight and no-hypertension showed more likely to amputation. Early detection of comorbidities is recommended to prevent amputation among patients with DFU.

## Introduction

Diabetic foot ulcer (DFU) is one of the most severe and costly complications of diabetes mellitus, leading to infection, lower-extremity amputation, and premature mortality [3,5]. This condition affects people with diabetes worldwide and represents a major public health challenge [1–3]. Globally, more than 537 million people are living with diabetes, and it is estimated that 19–34% will develop a DFU during their lifetime [4,5]. DFU accounts for approximately 20% of diabetes-related hospital admissions and is the leading cause of non-traumatic lower-limb amputations worldwide [6,7]. Approximately 20% of individuals with DFU undergo lower-extremity amputation, and nearly 10% die within one year of ulcer diagnosis [5–7].

The burden of DFU is particularly high in low- and middle-income countries, including Indonesia, where the prevalence of diabetes has increased substantially over the past decade. Indonesia ranks among the top ten countries globally for diabetes prevalence, with an estimated 19.5 million adults living with diabetes [4,8]. Hospital-based studies from Indonesia indicate that DFU affects a significant proportion of people with diabetes and contributes substantially to prolonged hospitalization, high treatment costs, disability, and preventable amputations. Despite this growing burden, national-level data on DFU outcomes, particularly post-amputation survival, remain limited.

DFU is associated with considerable morbidity and mortality. Severe infection, peripheral neuropathy, peripheral arterial disease, and delayed access to specialized foot care often associated with major or minor amputations. Evidence suggests that survival following DFU-related amputation is poor: pooled estimates indicate a 5-year mortality rate exceeding 50% among individuals with DFU who undergo amputation [9]. Cohort studies further demonstrate markedly reduced long-term survival among those undergoing major amputation compared with individuals whose ulcers heal without amputation [10,11]. These deaths are largely attributable to coexisting conditions such as cardiovascular disease, chronic kidney disease, and advanced peripheral arterial disease.

Although advances in diabetes management have improved glycemic control and ulcer care, substantial gaps remain in understanding long-term survival and prognostic factors following DFU-related amputation, particularly in Indonesia. Identifying incidence rates, survival patterns, and modifiable risk factors is essential to guide clinical decision-making, improve post-amputation care, and reduce preventable mortality. Therefore, this study aimed to determine the incidence rate and survival outcomes of amputation and to identify factors associated with survival among people with diabetic foot ulcers in Indonesia.

## Materials and methods

### Study design

This study employed a retrospective cohort design using medical record data from patients with diabetic foot ulcers (DFU) treated at Margono Hospital, Central Java, Indonesia, between 1 July 2019 and 30 June 2024.

### Study area

Margono Hospital is a tertiary public referral hospital located in Central Java, Indonesia, with a specialized diabetes care unit. The hospital receives referrals from primary and secondary healthcare facilities across the region and maintains standardized diabetes and wound-care protocols. Electronic medical records are routinely used for clinical documentation, enabling longitudinal follow-up of patients with DFU.

### Study population, inclusion and exclusion criteria

All adult patients (≥18 years) with type 2 diabetes mellitus who presented to Margono Hospital with a clinically diagnosed diabetic foot ulcer during the study period were eligible for inclusion. Both patients presenting with a newly diagnosed DFU and those referred with chronic or non-healing DFU were included. Patients were excluded if they had incomplete or missing electronic medical records, had a documented diagnosis of human immunodeficiency virus (HIV) infection or hemophilia, and underwent lower-extremity amputation prior to their first DFU-related presentation at Margono Hospital. Patients with a history of previous amputation before the current DFU episode were included, provided that no amputation had occurred for the index ulcer prior to hospital presentation. A total sampling approach was applied, resulting in 878 eligible DFU cases.

### Outcome definition and follow-up

The primary outcome was time to first lower-extremity amputation following DFU diagnosis. Follow-up time (in years) was calculated from the date of first DFU-related presentation to Margono Hospital, which was considered the date of diagnosis for this study, until the occurrence of amputation (event), death, loss to follow-up, ulcer healing, or the end of the study period. The maximum follow-up period was five years. Patients who died, were lost to follow-up, or achieved ulcer healing without amputation were treated as censored observations.

### Exposure variables

Body mass index (BMI) was classified using the World Health Organization (WHO) Asia-Pacific criteria [12]. BMI was included as an exposure variable because previous studies have reported associations between nutritional status, wound healing capacity, and risk of lower-extremity amputation among patients with diabetic foot ulcers [36–40]. DFU severity was assessed using the Wagner classification system and regrouped into three categories: (1) Grades 0–1, (2) Grades 2–3, and (3) Grades 4–5 [2]. Risk of foot ulceration was classified according to the International Working Group on the Diabetic Foot (IWGDF) 2019 guidelines. Patients were categorized as high risk if they had loss of protective sensation (LOPS) or peripheral arterial disease (PAD) in combination with a prior history of foot ulcer, lower-extremity amputation, or end-stage renal disease. LOPS was assessed using a 10-g Semmes–Weinstein monofilament and/or a 128-Hz tuning fork, while PAD was assessed using ankle–brachial index (ABI) measurements or Doppler ultrasound [13].

### Data collection

Data were extracted from electronic medical records by trained research assistants and reviewed for accuracy. The researcher verified the dataset with medical practitioners before analysis. Prior to statistical analysis, the data were revalidated by a healthcare professional to ensure integrity and additional verification and error correction were performed by the researcher. The data collection procedure was designed in 2024 and took up to 3 months. Training for enumerators was conducted in June 2024, while data collection began in July 2024. Subsequently, data collection was conducted from July 2024 to October 2024. Data were accessed for research purposes on 26/06/2024. Information on duration of diabetes, glycemic control indicators (e.g., HbA1c), smoking status, alcohol use, socioeconomic indicators, infection markers, serum albumin, and hemoglobin levels was not consistently available in the electronic medical records and therefore could not be included in the analysis.

### Statistical analysis

The baseline characteristics of DFU patients were described using frequency and percentage for categorical data. The mean, standard deviation (SD), median, and minimum and maximum ranges were used for continuous data. The incidence rate of DFU Amputation per 100 person-years since diagnosis and its 95% confidence interval (CI) were calculated using the Poisson distribution assumption. The IR of DFU amputation was calculated as the number of DFU amputations divided by the total person-years.

Kaplan-Meier methods were used to assess the survival rate from DFU diagnosis until amputation. IRs and survival rates of DFU amputation were reported for the 5-year follow-up. Simple cox regression analysis was used to evaluate the association between each factor and DFU survival one at a time, with results presented as crude hazard ratios (CHR). Independent variables for the initial model were selected based on plausibility, prior evidence, data completeness or a Wald test p-value < 0.25 in bivariate analysis [14]. Multiple cox regression analysis was performed to evaluate the association between each factor and DFU survival, adjusting for all factors. Results were presented as Adjusted Hazard Ratio and their 95%CI as well as the p-value. All tests were two-sided, with a statistically significant p-value of less than 0.05. Analyses were conducted using STATA version 18 (StataCorp, College Station, TX).

### Ethical consideration

The study protocol was reviewed and approved by the Ethics Committee of RSUD Prof. Dr. Margono Soekarjo Purwokerto (Margono Hospital), Indonesia, on 26 June 2024 (Approval No. 420/05089), and by the Center for Ethics in Human Research, Khon Kaen University, Thailand (Reference No. HE682022). As this was a retrospective analysis of existing medical records, no direct patient contact occurred. All data were fully anonymized prior to analysis. The requirement for informed consent was waived by both ethics’ committees.

## Results

### Characteristics of DFU patients

**Fig 1:**
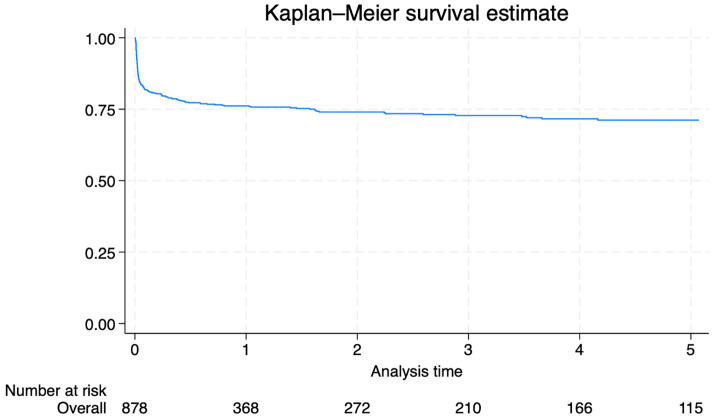
Kaplan-Meier curve of the chance of being amputated among T2DM patients with DFU.

**Fig 2:**
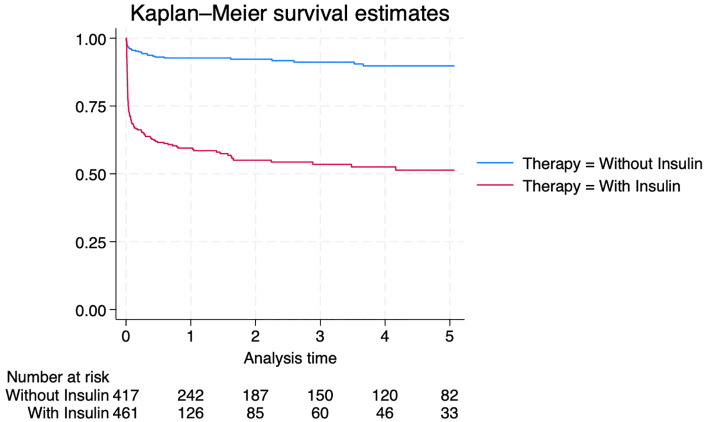
Kaplan-Meier curve of the chance of being amputated by Therapy among T2DM patients.

**Fig 3:**
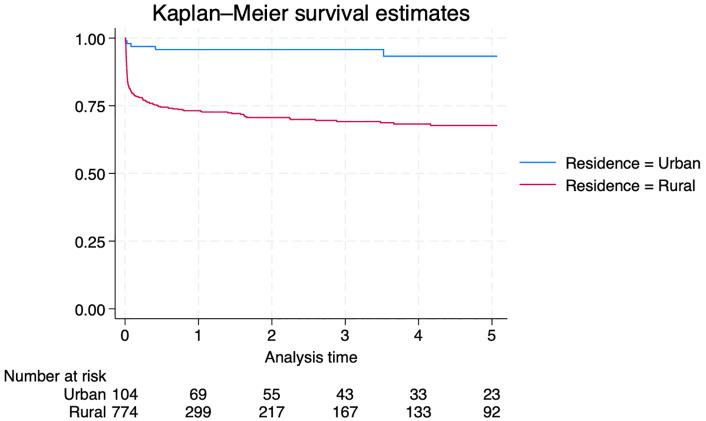
Kaplan-Meier curve of the chance of being amputated by Residence among T2DM patients.

A total of 878 patients with DFU were included in the analysis. During the observation period, 192 amputations were recorded, corresponding to 1,350.55 person-years of follow-up. This indicates that approximately one in five DFU patients eventually required amputation during the study period. Amputations were more frequent among females (54.7%) and older patients (≥60 years, 52.6%), with nearly all cases occurring among rural residents (97.4%). Most amputations were observed among patients with normal BMI (82.8%), those receiving insulin therapy (83.9%), and particularly those presenting with gangrene (84.9%). Renal disease accounted for 17.2% of amputations, while hypertension, cardiovascular disease, and neuropathy contributed smaller proportions. The overall IR of amputation was 14.22 per 100 person-years (95% CI: 12.34–16.38). The highest incidence was observed among patients with gangrene, reaching 85.68 per 100 person-years (95% CI: 73.49–99.90) (Table 1 and Figs 1–3).

**Table 1:** Incidence rates of lower-extremity amputation per 100 person-years with 95% confidence intervals among patients with diabetic foot ulcer (n = 878).

Characteristics	Amputation		(Person-Year)	IR/100 PY
	No (%)	Yes (%)		
**Overall**	686 (78.13)	192 (21.87)	1,350.55	14.22 (12.34-16.38)
**Sex**				
Male	317 (46.21)	87 (45.31)	649.79	13.39 (10.85-16.52)
Female	369 (53.79)	105 (54.69)	700.77	14.98 (12.38-18.14)
**Age (years)**				
< 60	334 (48.69)	91 (47.40)	644.51	14.12 (11.50-17.34)
≥ 60	352 (51.31)	101 (52.60)	706.04	14.31 (11.77-17.39)
Mean (SD)	60.30 ( )	60.84 ( )		
Median (Min: Max)	60.28 (8.38:92.61)	60.45 (38.69:86.47)		
**Residence**				
Urban	99 (14.43)	5 (2.60)	256.24	1.95 (0.81-4.69)
Rural	587 (85.57)	187 (97.40)	1,094.31	17.09 (14.81-19.72)
**Payment**				
BPJS	592 (86.30)	165 (85.94)	1,159.85	14.23 (12.21-16.57)
Non-BPJS	94 (13.70)	27 (14.06)	190.70	14.16 (9.71-20.65)
**BMI**				
Underweight and Normal weight	443 (64.58)	159 (82.81)	838.38	18.97 (16.24-22.15)
Overweight and obese	243 (35.42)	33 (17.19)	512.18	6.44 (4.58-9.06)
**T2DM Therapy**				
Without Insulin	386 (56.27)	31 (16.15)	907.90	3.41 (2.40-4.86)
With Insulin	300 (43.73)	161 (83.85)	442.66	36.37 (31.17-42.45)
**Status of Ulcer Risk**				
High Risk	118 (17.20)	42 (21.88)	220.38	19.06 (14.08-25.79)
Non-High Risk	568 (82.80)	150 (78.12)	1,130.17	13.27 (11.31-15.58)
**Severity of DFU**				
Gangrene	97 (14.14)	163 (84.90)	190.24	85.68 (73.49-99.90)
Non-Gangrene	589 (85.86)	29 (15.10)	1,160.31	2.50 (1.74-3.60)
**Comorbidity**				
Renal Disease				
Yes	89 (12.97)	33 (17.19)	162.58	20.30 (14.43-28.55)
No	597 (87.03)	159 (82.81)	1,187.98	13.38 (11.46-15.63)
**Hypertension**				
Yes	211 (30.76)	35 (18.23)	407.30	8.59 (6.17-11.97)
No	475 (69.24)	157 (81.77)	943.25	16.64 (14.23-19.46)
**CVD**				
Yes	83 (12.10)	4 (2.08)	197.89	2.02 (0.76-5.39)
No	603 (87.90)	188 (97.92)	1,152.66	16.31 (14.14-18.82)
**Neuropathy**				
Yes	137 (19.97)	12 (6.25)	329.53	3.64 (2.07-6.41)
No	549 (80.03)	180 (93.75)	1,021.02	17.63 (15.23-20.40)

DFU = Diabetic Foot Ulcer; IR = Incidence Rate; PY = Person-Year, CI = Confidence Interval; SD = Standard Deviation; Min = Minimum; Max = Maximum; BPJS = Indonesia National Health Insurance Scheme; BMI = Body Mass Index; T2DM = Type 2 Diabetes Mellitus; CVD = cardiovascular disease

Overall, the amputation-free survival rate among patients with DFU declined progressively over time, from 76.14% (95%CI: 72.82–79.11) at one year to 72.77% (95%CI: 69.08–76.10) at three years and 71.18% (95%CI: 67.22–74.75) at five years. Subgroup analyses demonstrated that the highest five-year survival rates were observed among patients with cardiovascular disease (94.37%, 95% CI: 85.17–97.93), urban residents (93.30%, 95% CI: 83.48–97.37), and patients with non-gangrene ulcers (92.87%, 95% CI: 89.62–95.13). In contrast, the lowest survival rates were observed in patients with gangrene (23.48%, 95% CI: 16.90–30.70), those receiving insulin therapy (51.37%, 95% CI: 44.81–57.53), those with high-risk of ulcer (63.02%, 95% CI: 52.35–71.94). These findings underscore the critical influence of ulcer severity, comorbidities, glycemic management, and healthcare access on long-term survival outcomes (Table 2).

**Table 2:** Amputation-free survival rates at 1-year, 3-year, and 5-year among patients with diabetic foot ulcer.

Characteristics	Survival Rate (%)						p-value
	1-Year		3-Year		5-Year		
	n (%)	95%CI	n (%)	95%CI	n (%)	95%CI	
**Overall**	**369 (76.14)**	**72.82-79.11**	**211 (72.77)**	**69.08-76.1**	**116 (71.18)**	**67.22-74.75**	
**Sex**							0.908
Male	171 (76.31)	71.27-80.59	107 (73.25)	67.78-77.94	58 (71.71)	65.89-76.70	
Female	199 (76.04)	71.46-79.99	105 (72.34)	67.12-76.88	59 (70.71)	65.07-75.61	
**Age (years)**							0.958
< 60	171 (76.20)	71.28-80.40	100 (72.67)	67.26-77.35	69 (72.67)	67.26-77.35	
≥ 60	199 (76.14)	71.46-80.16	112 (72.91)	67.68-77.44	48 (69.68)	63.69-74.89	
**Residence**							**<0.001**
Urban	70 (95.76)	89.07-98.39	44 (95.76)	89.07-98.39	24 (93.30)	83.48-97.37	
Rural	300 (73.18)	69.47-76.51	168 (69.16)	65.01-72.93	93 (67.72)	63.31-71.72	
**Payment**							0.691
BPJS	321 (76.36)	72.77-79.54	179 (72.46)	68.41-76.07	95 (71.01)	66.69-74.87	
Non-BPJS	49 (74.95)	65.26-82.29	33 (74.95)	65.26-82.29	22 (72.53)	61.79-80.71	
**BMI**							**<0.001**
Underweight and normal weight	221 (70.22)	65.84-74.15	132 (66.63)	61.83-70.97	80 (64.33)	59.16-69.02	
Overweight and obese	149 (88.02)	83.18-91.54	80 (85.15)	79.49-89.36	37 (85.15)	79.49-89.36	
**T2DM Therapy**							**<0.001**
Without Insulin	243 (92.70)	89.43-94.98	151 (91.16)	87.37-93.86	83 (89.80)	85.45-92.90	
With Insulin	127 (59.49)	54.06-64.51	61 (53.49)	47.39-59.20	34 (51.37)	44.81-57.53	
**Status of Ulcer Risk**							0.233
High Risk	63 (72.41)	63.63-79.41	33 (63.02)	52.35-71.94	18 (63.02)	52.35-71.94	
Non-High Risk	307 (76.95)	73.31-80.16	179 (74.79)	70.88-78.26	99 (72.85)	68.54-76.66	
**Severity of DFU**							**<0.001**
Gangrene	56 (33.86)	27.58-40.24	24 (27.27)	20.87-34.04	12 (23.48)	16.90-30.70	
Non-Gangrene	314 (95.25)	92.91-96.83	188 (93.51)	90.62-95.54	105 (92.87)	89.62-95.13	
**Renal Disease**							0.137
Yes	44 (67.62)	56.87-76.24	25 (63.42)	51.70-73.03	17 (63.42)	51.70-73.03	
No	326 (77.42)	73.92-80.52	187 (74.20)	70.30-77.67	100 (72.35)	68.10-76.13	
**Hypertension**							**<0.001**
Yes	129 (84.47)	78.78-88.75	58 (82.12)	75.46-87.12	17 (82.12)	75.46-87.12	
No	241 (72.54)	68.38-76.25	154 (68.68)	64.11-72.79	100 (66.72)	61.86-71.11	
**CVD**							**<0.001**
Yes	57 (96.42)	89.29-98.83	29 (94.37)	85.17-97.93	19 (94.37)	85.17-97.93	
No	313 (73.55)	69.9-76.83	183 (70.01)	65.97-73.66	98 (68.23)	63.91-72.16	
**Neuropathy**							**<0.001**
Yes	104 (94.94)	89.67-97.56	48 (89.38)	80.89-94.23	18 (87.20)	77.33-92.97	
No	266 (71.49)	67.55-75.04	164 (68.62)	64.38-72.47	99 (67.19)	62.71-71.26	

DFU = Diabetic Foot Ulcer; IR = Incidence Rate; CI = Confidence Interval; BPJS = Indonesia National Health Insurance Scheme; BMI = Body Mass Index; T2DM = Type 2 Diabetes Mellitus; CVD = cardiovascular disease; p-value: Chi Square

**Fig 4:**
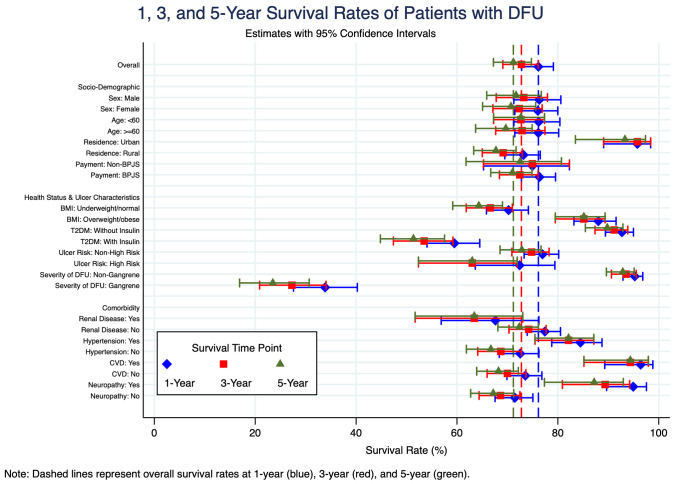
Forest plot survival rate and 95% confidence interval.

Fig 4 further illustrates these patterns through a forest plot of survival rates and their 95% confidence intervals across subgroups, highlighting the need for targeted interventions for high-risk populations (Fig 4).

### Factors associated with amputation

The analysis using simple Cox regression revealed potential factors associated with amputation risk (p < 0.25). These included rural residence (CHR: 6.54; 95% CI: 2.69–15.91), having an underweight or normal BMI compared to overweight/obese (CHR: 2.64; 95% CI: 1.82–3.85), use of insulin therapy (CHR: 6.66; 95% CI: 4.52–9.8), and presence of high-risk ulcer status (CHR: 1.23; 95% CI: 0.87–1.73). The strongest association was found for gangrene (CHR: 20.52; 95% CI: 13.78–30.55). Regarding comorbidities, the absence of renal disease showed a potential association (CHR: 0.75; 95% CI: 0.52–1.1). Furthermore, the absence of hypertension (CHR: 2.13; 95% CI: 1.48–3.08), absence of cardiovascular disease (CVD) (CHR: 6.72; 95% CI: 2.5–18.1), and absence of neuropathy (CHR: 4.30; 95% CI: 2.4–7.72) were also found to be significantly associated with amputation (Table 3).

**Table 3:** Factors associated with risk of amputation among patients with diabetic foot ulcer based on simple cox regression.

Factors	Total Sample	Person-Year	IR/100	Crude HR	95% CI	p-value
**Sex**						0.908
Male	404	649.79	13.39	1		
Female	474	700.77	14.98	1.02	0.77-1.35	
**Age (years)**						0.958
< 60	425	644.51	14.12	1		
≥ 60	453	706.04	14.31	1.01	0.76-1.34	
**Residence**						**<0.001**
Urban	104	256.24	1.95	1		
Rural	774	1,094.31	17.09	6.54	2.69-15.91	
**Payment**						0.693
Non-BPJS	121	190.70	14.16	1		
BPJS	757	1,159.85	14.23	0.92	0.61-1.38	
**BMI**						**<0.001**
Overweight and obese	276	512.18	6.44	1		
Underweight and normal weight	602	838.38	18.97	2.64	1.82-3.85	
**T2DM Therapy**						**<0.001**
Without Insulin	417	907.90	3.41	1		
With Insulin	461	442.66	36.37	6.66	4.52-9.8	
**Status of Ulcer Risk**						0.236
Non-High Risk	718	1,130.17	13.27	1		
High Risk	160	220.38	19.06	1.23	0.87-1.73	
**Severity of DFU**						**<0.001**
Non-Gangrene	618	1,160.31	2.50	1		
Gangrene	260	190.24	85.68	20.52	13.78-30.55	
**Renal Disease**						0.140
Yes	122	162.58	20.30	1		
No	756	1,187.98	13.38	0.75	0.52-1.1	
**Hypertension**						**<0.001**
Yes	246	407.30	8.59	1		
No	632	943.25	16.64	2.13	1.48-3.08	
**CVD**						**<0.001**
Yes	87	197.89	2.02	1		
No	791	1,152.66	16.31	6.72	2.5-18.1	
**Neuropathy**						**<0.001**
Yes	149	329.53	3.64	1		
No	729	1,021.02	17.63	4.30	2.4-7.72	

DFU = Diabetic Foot Ulcer; IR = Incidence Rate; CI = Confidence Interval; SD = Standard Deviation; Min = Minimum; Max = Maximum; BPJS = Indonesia National Health Insurance Scheme; BMI = Body Mass Index; T2DM = Type 2 Diabetes Mellitus; CVD = cardiovascular disease; HR = Hazard Ratio

Using multiple Cox regression, after adjusting for potential confounders and checking for multicollinearity, several factors remained significantly associated with amputation. Patients receiving insulin therapy had more than five times the risk of amputation compared with those not on insulin (AHR: 5.51; 95% CI: 3.73–8.14). Residing in rural areas was also strongly associated with increased risk (AHR: 4.14; 95% CI: 1.70–10.12). Moreover, patients without neuropathy (AHR: 2.40; 95% CI: 1.30–4.42), those with underweight or normal BMI (AHR: 2.04; 95% CI: 1.40–2.98), and those without hypertension (AHR: 1.52; 95% CI: 1.03–2.22) were at higher risk of amputation (Table 4).

**Table 4:** Factors associated with risk of amputation among patients with diabetic foot ulcer based on multiple cox regression.

Factors	Total Sample	Person-Year	IR/100	Crude HR	Adj. HR	95% CI	p-value
**T2DM Therapy**							<0.001
Without Insulin	417	907.90	3.41	1	1		
With Insulin	461	442.66	36.37	6.66	5.51	3.73-8.14	
**Residence**							0.002
Urban	104	256.24	1.95	1	1		
Rural	774	1,094.31	17.09	6.54	4.14	1.70-10.12	
**Neuropathy**							0.005
Yes	149	329.53	3.64	1	1		
No	729	1,021.02	17.63	4.30	2.40	1.30-4.42	
**BMI**							<0.001
Overweight and obese	276	512.18	6.44	1	1		
Underweight and normal weight	602	838.38	18.97	2.64	2.04	1.40-2.98	
**Hypertension**							0.033
Yes	246	407.30	8.59	1	1		
No	632	943.25	16.64	2.13	1.52	1.03-2.22	

DFU = Diabetic Foot Ulcer; IR = Incidence Rate; CI = Confidence Interval; BMI = Body Mass Index; T2DM = Type 2 Diabetes Mellitus; CVD = cardiovascular disease; HR = Hazard Ratio; Adj. HR = Adjusted Hazard Ratio

## Discussions

In this study, the overall incidence rate of amputation among patients with diabetic foot ulcers was 14.22 per 100 person-years (95% CI: 12.34–16.38), with the highest incidence observed among patients with gangrene, reaching 85.68 per 100 person-years (95% CI: 73.49–99.90). This finding is relatively consistent with previous reports, which documented an amputation rate of 9.9% in China [15] and 14.11% in Surabaya, Indonesia [16]. This discrepancy may be attributed to differences in follow-up duration, definitions of DFU severity, healthcare access, and the higher proportion of gangrene cases in our study cohort, highlighting how the risk of amputation remains substantial, particularly for patients presenting with severe ulceration or gangrene.

Our one-year, three-year, and five-year survival rates (76.14%, 72.77%, and 71.18%, respectively) were slightly lower than the 85.7% three-year survival reported in an earlier Indonesian study [17], and lower than the 81.2% one-year survival found in other studies. However, our five-year survival was considerably higher than the 49.7% reported previously [18]. These differences could be explained by differences in prevalence of comorbidities, variation in DFU management protocols, or better post-amputation care in our setting.

Patients receiving insulin therapy had a significantly higher risk of amputation compared to those not on insulin, reflecting lower survival among insulin users in our cohort. This finding aligns with previous evidence suggesting that insulin use often indicates more severe diabetes or poor glycemic control, highlighting its potential role as a proxy for disease severity [19]. Supporting this, a study employing multiple logistic regression found that insulin therapy was associated with developing diabetic foot ulceration compared to oral hypoglycemic agents (OHA) [19]. Beyond ulceration, insulin use has also been linked to an elevated risk of major adverse cardiovascular events (MACE) and hospitalization for heart failure in patients with type 2 diabetes and acute coronary syndrome [20]. Poor adherence to insulin therapy may exacerbate hyperglycemia, a known risk factor for diabetic foot complications [21]. Effective self-care practices are essential for preventing such complications, and previous studies have demonstrated that self-care scores vary significantly depending on the type of diabetes treatment [22]. Moreover, an institution-based unmatched case-control study reported a positive association between insulin monotherapy and diabetic foot ulcers [23]. Together, these findings reinforce the importance of individualized diabetes management and careful monitoring of patients on insulin to prevent foot complications.

Residing in rural areas was associated with a fourfold increased risk of amputation compared to urban residents, consistent with prior evidence indicating that limited healthcare access and delayed medical intervention in rural settings contribute to adverse outcomes. This observation underscores persistent systemic disparities in healthcare delivery across low- and middle-income countries. Empirical data have shown that rural residence is strongly associated with the development of diabetic foot ulcers, suggesting that rural patients are approximately twice as likely to develop foot ulcers comparing with their urban counterparts [24]. Such disparities are partly attributable to suboptimal glycemic, blood pressure, and lipid control among rural populations relative to urban populations [25]. Furthermore, the scarcity of healthcare facilities and trained personnel in rural areas contributes to delays in diagnosing and managing chronic conditions, including diabetes, thereby increasing the likelihood of complications such as DFUs [26,27]. Mental health comorbidities, particularly depression, which is more prevalent in rural populations, may further impair adherence to self-care practices, including diabetic foot care, thereby elevating the risk of DFU recurrence [28,29]. Additionally, rural living conditions, which often constrain access to nutritious food and opportunities for physical activity, can adversely affect diabetes management and the prevention of DFUs [30]. Collectively, these factors explain the heightened vulnerability of rural residents to adverse diabetic foot outcomes and emphasize the need for targeted interventions to mitigate these risks.

Interestingly, patients without neuropathy demonstrated a higher risk of amputation compared to those with neuropathy, which contrasts with previous studies linking these comorbidities to poorer outcomes [31]. This could reflect more intensive routine care among patients with established comorbidities, facilitating earlier detection and management of complications, whereas patients without recognized comorbidities may have underdiagnosed or inadequately managed conditions. Specifically, diabetic foot ulcers are often identified earlier in patients with comorbidities due to more frequent healthcare encounters, which may represent an incidental benefit of routine monitoring for other chronic conditions [32,33]. Comorbidities such as diabetic neuropathy and peripheral arterial disease remain well-established predictors of DFUs, necessitating closer surveillance by healthcare providers [33]. Furthermore, patients managing multiple health conditions may exhibit heightened awareness of physical changes, particularly related to foot health, thereby promoting early symptom reporting and proactive self-care behaviors [34]. Emerging evidence also suggests that biomarkers, including miR-204-3p, are associated with the onset and resolution of DFUs, highlighting the potential for early detection in patients with comorbidities through biomarker monitoring [35]. Socio-economic determinants, such as lower socio-economic position, have been associated with higher DFU incidence; however, they may also lead to increased healthcare interactions, indirectly facilitating earlier identification and management of foot ulcers [32]. These findings emphasize that the apparent protective effect observed in patients with neuropathy may be attributed to heightened surveillance and proactive management rather than the absence of risk.

Underweight and normal-weight patients exhibited a twofold higher risk of amputation compared to overweight or obese individuals. Rather than indicating a protective effect of obesity per se, this finding may reflect the adverse impact of undernutrition, frailty and reduced physiological reserve on wound healing and infection control in patients with diabetic foot ulcers. Lower body mass has been associated with greater DFU severity and poorer healing outcomes, suggesting that inadequate nutritional status may exacerbate ulcer progression and increase the likelihood of amputation [36].Malnutrition is also linked to systemic inflammation, immune dysfunction, and higher mortality in underweight individuals with chronic disease, which may further compromise recovery in patients with DFU [37,38]. Although some studies in chronic illness populations describe an “obesity paradox,” whereby overweight or moderately obese individuals experience improved survival, evidence supporting this phenomenon specifically for DFU-related amputation remains limited and should be interpreted with caution [37,39]. Additionally, deficiencies in essential micronutrients including vitamin D, vitamin C, magnesium, and selenium are common among patients with DFU and have been shown to impair wound healing processes [40]. These findings underscore the importance of comprehensive nutritional assessment and individualized nutritional interventions as part of multidisciplinary DFU management. Future prospective studies are warranted to clarify the causal relationship between BMI, nutritional status, and amputation risk in patients with diabetic foot ulcers.

The finding that patients without hypertension had worse survival outcomes is somewhat contradictory to expectations. One explanation might be that hypertensive patients are more regularly monitored and treated, leading to better overall management of diabetic complications including DFU. Because of its relationship to vascular health and diabetes regulation, effective blood pressure control is crucial for DFU outcomes [41–43]. Recent studies show a more complex association between hypertension and amputation risk in DFU patients. Diabetes and hypertension often affect each other’s treatment. Diabetes drugs including GLP-1 receptor agonists and SGLT2 inhibitors reduce blood pressure. This suggests that diabetes treatment can also reduce hypertension, making therapy choices helpful for both illnesses [44]. Maintaining blood pressure below 130/80 mmHg through pharmacologic therapy, lifestyle modification, and agents such as ACE inhibitors or ARBs is recommended, particularly in patients with resistant hypertension [45]. While hypertension awareness does not directly prevent DFUs, it promotes optimized cardiovascular and metabolic management, thereby reducing ulcer risk [46].

This study provides long-term evidence on survival and amputation outcomes in patients with diabetic foot ulcers, while uncovering paradoxical associations with neuropathy, hypertension, and BMI, as well as the strong influence of rural residence. As an endocrine disease, diabetes can affect the development and management of comorbidities such as hypertension and neuropathy, which in turn influence DFU outcomes, making it difficult to determine the causal direction between diabetes, comorbidities, and ulcer progression. These findings emphasize the interplay between biological, clinical, and social determinants, highlighting the need for individualized management and targeted interventions to reduce the burden of diabetic foot complications.

### Strength and limitation of the study

This study has several strengths. It included a large cohort of patients with diabetic foot ulcers followed for up to five years, with minimal missing data. The study was conducted at Margono Hospital in Central Java, one of the largest public hospitals in Indonesia with a specialized diabetes department, and included a substantial number of referred DFU cases, allowing for robust survival and incidence analyses. Nevertheless, several limitations should be acknowledged. First, the findings may not be fully generalizable to the entire Indonesian population, as the study was conducted at a single tertiary hospital located on Java Island. Second, information on deaths occurring outside the hospital was unavailable, which may have led to underestimation or misclassification of survival outcomes. Third, important clinical and behavioral variables including duration of diabetes, glycemic control, smoking status, alcohol consumption, infection status, anemia, and nutritional biomarkers such as serum albumin were unavailable or incompletely recorded in the medical records. The absence of these variables may have resulted in residual confounding. Despite these limitations, this study provides valuable evidence on the incidence and survival outcomes of amputation among patients with diabetic foot ulcers in Indonesia. Future multicenter and prospective studies across different regions of the country are warranted to confirm and extend these findings.

## Conclusions

The overall IR of amputation among patients with DFU in Indonesia was high, with the highest rates observed among patients with gangrene. The survival rate declined progressively from one year to five years. The overall 5-year survival rate among DFU patients in Indonesia was low, with the highest survival observed among patients with cardiovascular disease and the lowest among those with gangrene. Patients receiving insulin therapy, residing in rural areas, those without neuropathy, patients with underweight or normal BMI and those without hypertension were more likely to undergo amputation.

To mitigate amputation risk, DFU patients should undergo routine screening for neuropathy and hypertension, and early detection programs, particularly for rural populations, should be strengthened. Standardized hospital-based screening for high-risk patients, coupled with multidisciplinary management involving endocrinologists, vascular specialists, and wound care teams, is recommended. Patient education should emphasize early recognition of complications and timely medical intervention, with special attention to self-care and adherence to treatment. Mobile health units and telemedicine can improve access to diabetes and DFU care in rural areas, while ensuring affordable insulin and wound care supplies may further prevent severe outcomes. Future research should explore the biological and social determinants influencing DFU prognosis, including the paradoxical associations between amputation risk and absence of comorbidities, as well as the impact of early detection and intervention on patient outcomes.

## References

[1] Bundó M, Vlacho B, Llussà J, Bobé I, Aivar M, Ciria C, et al. Prediction of outcomes in subjects with type 2 diabetes and diabetic foot ulcers in Catalonian primary care centers: a multicenter observational study. J Foot Ankle Res. 2023;16(1):8. doi: 10.1186/s13047-023-00602-6 36849888 PMC9972716

[2] Wang X, Yuan C-X, Xu B, Yu Z. Diabetic foot ulcers: Classification, risk factors and management. World J Diabetes. 2022;13(12):1049–65. doi: 10.4239/wjd.v13.i12.1049 36578871 PMC9791567

[3] McDermott K, Fang M, Boulton AJM, Selvin E, Hicks CW. Etiology, Epidemiology, and Disparities in the Burden of Diabetic Foot Ulcers. Diabetes Care. 2023;46:209–11. doi: 10.2337/dci22-0043 36548709 PMC9797649

[4] IDF-International Diabetes Federation. The diabetic foot. Brussels, Belgium: International Diabetes Federation. 2020.

[5] Armstrong DG, Boulton AJM, Bus SA. Diabetic Foot Ulcers and Their Recurrence. New England Journal of Medicine. 2017;376:2367–75. doi: 10.1056/nejmra1615439 28614678

[6] Hoffstad O, Mitra N, Walsh J, Margolis DJ. Diabetes, lower-extremity amputation, and death. Diabetes Care. 2015;38(10):1852–7. doi: 10.2337/dc15-0536 26203063

[7] Meloni M, Izzo V, Giurato L, Lázaro-Martínez JL, Uccioli L. Prevalence, Clinical Aspects and Outcomes in a Large Cohort of Persons with Diabetic Foot Disease: Comparison between Neuropathic and Ischemic Ulcers. J Clin Med. 2020;9(6):1780. doi: 10.3390/jcm9061780 32521700 PMC7356179

[8] Liao X, Li S-H, El Akkawi MM, Fu X-B, Liu H-W, Huang Y-S. Surgical amputation for patients with diabetic foot ulcers: A Chinese expert panel consensus treatment guide. Front Surg. 2022;9:1003339. doi: 10.3389/fsurg.2022.1003339 36425891 PMC9679004

[9] Chen L, Sun S, Gao Y, Ran X. Global mortality of diabetic foot ulcer: A systematic review and meta-analysis of observational studies. Diabetes Obes Metab. 2023;25(1):36–45. doi: 10.1111/dom.14840 36054820

[10] Lo ZJ, Surendra NK, Saxena A, Car J. Clinical and economic burden of diabetic foot ulcers: A 5-year longitudinal multi-ethnic cohort study from the tropics. Int Wound J. 2021;18(3):375–86. doi: 10.1111/iwj.13540 33497545 PMC8244009

[11] Jupiter DC, Thorud JC, Buckley CJ, Shibuya N. The impact of foot ulceration and amputation on mortality in diabetic patients. I: From ulceration to death, a systematic review. Int Wound J. 2016;13(5):892–903. doi: 10.1111/iwj.12404 25601358 PMC7950078

[12] World Health Organization. International Association for the Study of Obesity, International Obesity Task Force. Australia: Australia. 2000.

[13] Bus SA, Lavery LA, Monteiro-Soares M, Rasmussen A, Raspovic A, Sacco ICN, et al. Guidelines on the prevention of foot ulcers in persons with diabetes (IWGDF 2019 update). Diabetes Metab Res Rev. 2020;36 Suppl 1:e3269. doi: 10.1002/dmrr.3269 32176451

[14] Hosmer D, Lemeshow S. Applied logistic regression. 2nd Edition ed. New York: Willey. 2000.

[15] Lu Q, Wang J, Wei X, Wang G, Xu Y. Risk Factors for Major Amputation in Diabetic Foot Ulcer Patients. Diabetes Metab Syndr Obes. 2021;14:2019–27. doi: 10.2147/DMSO.S307815 33976562 PMC8106455

[16] Hariftyani AS, Novida H, Edward M. Profile of Diabetic Foot Ulcer Patients at Tertiary Care Hospital in Surabaya, Indonesia. JBE. 2021;9(3):293. doi: 10.20473/jbe.v9i32021.293-302

[17] Yunir E, Hidayah CD, Harimurti K, Kshanti IAM. Three Years Survival and Factor Predicting Amputation or Mortality in Patients with High Risk for Diabetic Foot Ulcer in Fatmawati General Hospital, Jakarta. J Prim Care Community Health. 2022;13:1–7. doi: 10.1177/21501319211063707 PMC8744153 34986684

[18] Vuorlaakso M, Kiiski J, Salonen T, Karppelin M, Helminen M, Kaartinen I. Major Amputation Profoundly Increases Mortality in Patients With Diabetic Foot Infection. Front Surg. 2021;8:655902. doi: 10.3389/fsurg.2021.655902 33996886 PMC8120024

[19] Tola A, Regassa LD, Ayele Y. Prevalence and associated factors of diabetic foot ulcers among type 2 diabetic patients attending chronic follow-up clinics at governmental hospitals of Harari Region, Eastern Ethiopia: A 5-year (2013-2017) retrospective study. SAGE Open Med. 2021;9. doi: 10.1177/2050312120987385 33552513 PMC7838876

[20] Schwartz GG, Nicholls SJ, Toth PP, Sweeney M, Halliday C, Johansson JO, et al. Relation of insulin treatment for type 2 diabetes to the risk of major adverse cardiovascular events after acute coronary syndrome: an analysis of the BETonMACE randomized clinical trial. Cardiovasc Diabetol. 2021;20(1):125. doi: 10.1186/s12933-021-01311-9 34158057 PMC8218391

[21] Chan JCN, Gagliardino JJ, Ilkova H, Lavalle F, Ramachandran A, Mbanya JC, et al. One in Seven Insulin-Treated Patients in Developing Countries Reported Poor Persistence with Insulin Therapy: Real World Evidence from the Cross-Sectional International Diabetes Management Practices Study (IDMPS). Adv Ther. 2021;38(6):3281–98. doi: 10.1007/s12325-021-01736-4 33978906 PMC8189989

[22] Montazeri N, Bakhshi S, Malekzadeh R, Ziapour A, Chaboksavar F, Yazdi F, et al. Investigating the factors affecting the self-care behaviors of patients with type II diabetes and the role of demographic variables: A case study in Iran. J Educ Health Promot. 2023;12:291. doi: 10.4103/jehp.jehp_1307_22 37849857 PMC10578564

[23] Woldemariam GT, Atnafu NT, Radie YT, Wolde GT, Gebreagziabher TT, Gebrehiwot TG, et al. Determinants of Diabetic Foot Ulcer Among Adult Patients with Diabetes Attending the Diabetic Clinic in Tikur Anbessa Specialized Hospital, Addis Ababa, Ethiopia: Unmatched Case-Control Study. Diabetes Metab Syndr Obes. 2020;13:3739–47. doi: 10.2147/DMSO.S265988 33116723 PMC7571574

[24] Mariam TG, Alemayehu A, Tesfaye E, Mequannt W, Temesgen K, Yetwale F. Prevalence of diabetic foot ulcer and associated factors among adult diabetic patients who attend the diabetic follow-up clinic at the University of Gondar Referral Hospital, North West Ethiopia, 2016: Institutional-based cross-sectional study. J Diabetes Res. 2017. doi: 10.1155/2017/2879249 PMC5534295 28791310

[25] HPACC rural-urban appendices. 2022.

[26] Lavoie CA, Voaklander D, Beach JR, Gross DP. The association between rurality and return to work for workers’ compensation claimants with work-related musculoskeletal injuries: An analysis of workers who failed to return to work within typical healing time frames. Int J Occup Med Environ Health. 2017;30(5):715–29. doi: 10.13075/ijomeh.1896.00926 28584330

[27] Ipsen C, Ward B, Myers A. Events Across the Life Course Contribute to Higher Mobility Impairment Rates in Rural U.S. Frontiers in Rehabilitation Sciences. 2022;3. doi: 10.3389/fresc.2022.863716 PMC9397967 36188967

[28] Roopsawang I, Aree-Ue S, Baurangthienthong S, Boontham J, Phiboonleetrakun Y. Path Model Factors Associated with Depressive Symptoms among Older Thais Living in Rural Areas. Geriatrics (Basel). 2022;7(3):69. doi: 10.3390/geriatrics7030069 35735774 PMC9222783

[29] Kim S, Cho S, Morgan MR. Neighborhood and Depressive Symptoms in Older Adults Living in Rural and Urban Regions in South Korea. Healthcare (Basel). 2023;11(4):476. doi: 10.3390/healthcare11040476 36833010 PMC9957275

[30] T. M, D. S, G. P. Analysis of risk factors for cardiovascular disease among adults living in rural areas of India: A survey. Cardiometry. 2022;320–5. doi: 10.18137/cardiometry.2022.24.320325

[31] Sen S, Barsun A, Romanowski K, Palmieri T, Greenhalgh D. Neuropathy May Be an Independent Risk Factor for Amputation After Lower-Extremity Burn in Adults With Diabetes. Clin Diabetes. 2019;37(4):352–6. doi: 10.2337/cd18-0066 31660008 PMC6794228

[32] Ha JH, Jin H, Park J-U. Association between socioeconomic position and diabetic foot ulcer outcomes: a population-based cohort study in South Korea. BMC Public Health. 2021;21(1):1395. doi: 10.1186/s12889-021-11406-3 34261483 PMC8281670

[33] Abuhay HW, Yenit MK, Wolde HF. Incidence and predictor of diabetic foot ulcer and its association with change in fasting blood sugar among diabetes mellitus patients at referral hospitals in Northwest Ethiopia. PLoS One. 2022;17. doi: 10.1371/journal.pone.0274754 PMC9560537 36227947

[34] Lewis HR, Chaplin WJ, Mcwilliams DF, Millar BS, Shahtaheri S, Gladman JFR. The association of painful and non-painful comorbidities with central mechanisms of knee pain. Ann Rheum Dis. 2022;81:1822.1–1822. doi: 10.1136/annrheumdis-2022-eular.1586

[35] Zhao X, Xu M, Tang Y, Xie D, Deng L, Chen M, et al. Decreased expression of miR-204-3p in peripheral blood and wound margin tissue associated with the onset and poor wound healing of diabetic foot ulcers. Int Wound J. 2023;20(2):413–29. doi: 10.1111/iwj.13890 35879811 PMC9885452

[36] Madmoli M, Madmoli Y, Taqvaeinasab H, Khodadadi M, Darabiyan P, Rafi A. Some influential factors on severity of diabetic foot ulcers and Predisposing of limb amputation: A 7-year study on diabetic patients. IJAM. 2019;10(1):75–81. doi: 10.47552/ijam.v10i1.1222

[37] Li S, Zhang W, Fu Z, Liu H. Impact of obesity on all-cause and cause-specific mortality among critically ill men and women: a cohort study on the eICU database. Front Nutr. 2023;10:1143404. doi: 10.3389/fnut.2023.1143404 37153915 PMC10160369

[38] Kiss N, Prado CM, Daly RM, Denehy L, Edbrooke L, Baguley BJ, et al. Low muscle mass, malnutrition, sarcopenia, and associations with survival in adults with cancer in the UK Biobank cohort. J Cachexia Sarcopenia Muscle. 2023;14(4):1775–88. doi: 10.1002/jcsm.13256 37212184 PMC10401543

[39] Masoudkabir F, Yavari N, Jameie M, Pashang M, Sadeghian S, Salarifar M, et al. The association between different body mass index levels and midterm surgical revascularization outcomes. PLoS One. 2022;17(9):e0274129. doi: 10.1371/journal.pone.0274129 36174074 PMC9522296

[40] Kurian SJ, Baral T, Unnikrishnan MK, Benson R, Munisamy M, Saravu K. The association between micronutrient levels and diabetic foot ulcer: A systematic review with meta-analysis. Frontiers in Endocrinology. 2023. doi: 10.3389/fendo.2023.1152854 PMC10090454 37065742

[41] Doğruel H, Aydemir M, Balci MK. Management of diabetic foot ulcers and the challenging points: An endocrine view. World J Diabetes. 2022;13(1):27–36. doi: 10.4239/wjd.v13.i1.27 35070057 PMC8771264

[42] Akkus G, Sert M. Diabetic foot ulcers: A devastating complication of diabetes mellitus continues non-stop in spite of new medical treatment modalities. World J Diabetes. 2022;13(12):1106–21. doi: 10.4239/wjd.v13.i12.1106 36578865 PMC9791571

[43] Raja JM, Maturana MA, Kayali S, Khouzam A, Efeovbokhan N. Diabetic foot ulcer: A comprehensive review of pathophysiology and management modalities. World J Clin Cases. 2023;11(8):1684–93. doi: 10.12998/wjcc.v11.i8.1684 36970004 PMC10037283

[44] Naha S, Gardner MJ, Khangura D, Kurukulasuriya LR, Sowers JR. Hypertension in Diabetes. 2000.

[45] Gunes Y. A brief approach to hypertension in type 2 diabetes mellitus. Explor Endocr Metab Dis. 2025. doi: 10.37349/eemd.2025.101422

[46] Wang X, Xu M, Meng L, Song M, Jia Z, Zhao L, et al. The awareness and determinants of diabetic foot ulcer prevention among diabetic patients: Insights from NHANES (2011-2018). Prev Med Rep. 2023;36:102433. doi: 10.1016/j.pmedr.2023.102433 37781107 PMC10534215

