# Supplementary material for: Survival analysis of time to amputation following diabetic foot ulcer diagnosis among Indonesians
Source: PLoS One. 2026 Jun 16;21(6):e0343399. doi: 10.1371/journal.pone.0343399 (PMC13271469; doi:10.1371/journal.pone.0343399)
Supplement: Attachment — Submitted filename: Response to editor comments.docx [file pone.0343399.s001.docx]

**Journal Name**: **PLOS ONE**

**Manuscript ID:** PONE-D-25-51099

**Title:** Survival Analysis of Time to Amputation following Diabetic Foot Ulcer Diagnosis among Indonesians

Dear Editor,

Thank you very much for your efforts on our manuscript. We are thankful for the editor’s critical comments and have revised the manuscript accordingly. The responses to editor comments are summarized in this letter point-by-point, and the corresponding revisions are made in the revised manuscript.

We appreciate your precious time in reviewing our manuscript, and we would be grateful if you would consider this manuscript for publication after review.

Yours sincerely,

Prof. Dr. Wongsa Laohasiriwong

Faculty of Public Health

Khon Kaen University

**Response to Editor**

Manuscript Entitled **“Survival Analysis of Time to Amputation following Diabetic Foot Ulcer Diagnosis among Indonesians”**

**Editor's comments**

**Comment 1.**  Please report in the Methods section the dates when data were accessed for research purposes in DD/MM/YYYY format. Please note that if this information is not included when your manuscript is resubmitted, it may be rejected.

**Response: We thank the editor for this valuable comment. In the revised manuscript, we have now included the date when data were accessed for research purposes, in DD/MM/YYYY format, as requested. Specifically, we added the following sentence at the end of the *Data Collection* subsection: “Data were accessed for research purposes on 26/06/2024.”**
