# Supplementary material for: Survival analysis of time to amputation following diabetic foot ulcer diagnosis among Indonesians
Source: PLoS One. 2026 Jun 16;21(6):e0343399. doi: 10.1371/journal.pone.0343399 (PMC13271469; doi:10.1371/journal.pone.0343399)
Supplement: Attachment — Submitted filename: Response to reviewer.docx [file pone.0343399.s002.docx]

**Response to Reviewer**

**Journal Name:** **PLOS One**

**Manuscript ID:** PONE-D-25-51099

**Title:** Survival Analysis of Time to Amputation following Diabetic Foot Ulcer Diagnosis among Indonesians

**Authors:** Lianawati^1^<https://orcid.org/0009-0005-8878-9409>, Wongsa Laohasiriwong^2*^<https://orcid.org/0000-0001-7838-716X>, Kyaw Min Htike^1^<https://orcid.org/0009-0004-7427-1425>, Kavin Thinkhamrop^2^<https://orcid.org/0000-0002-2727-2925>, Roshan Kumar Mahato^2^<https://orcid.org/0000-0001-9287-2743>

Dear Editor,

Thank you very much for your efforts on our manuscript. We are thankful for the editor and reviewer’s critical comments and have revised the manuscript accordingly. The responses to editor and reviewer comments are summarized in this letter point-by-point.

We appreciate your precious time in reviewing our manuscript, and we would be grateful if you would consider this manuscript for publication after review.

Yours sincerely,

Wongsa Laohasiriwong

Corresponding Author

Faculty of Public Health, Khon Kaen University, Thailand

**Response to Editor**

**Comment 1.** Please ensure that your manuscript meets PLOS ONE's style requirements, including those for file naming. The PLOS ONE style templates can be found at https://journals.plos.org/plosone/ s/file?id=wjVg/PLOSOne_formatting_sample_ main_body .pdf and https://journals.plos. org /plosone/s/file?id=ba62/PLOSOne_formatting _sample_ title_authors_affiliations.pdf

**Response: Thank you for this guidance. We have revised the manuscript to fully comply with PLOS ONE formatting and style requirements, including file naming, structure, headings, and layout, using the official PLOS ONE formatting templates for the main body and title/authors/affiliations. The revised files have been uploaded accordingly.**

**Comment 2.** When completing the data availability statement of the submission form, you indicated that you will make your data available on acceptance. We strongly recommend all authors decide on a data sharing plan before acceptance, as the process can be lengthy and hold up publication timelines. Please note that, though access restrictions are acceptable now, your entire data will need to be made freely accessible if your manuscript is accepted for publication. This policy applies to all data except where public deposition would breach compliance with the protocol approved by your research ethics board. If you are unable to adhere to our open data policy, please kindly revise your statement to explain your reasoning and we will seek the editor's input on an exemption. Please be assured that, once you have provided your new statement, the assessment of your exemption will not hold up the peer review process.

**Response: Thank you for this important clarification. We have carefully reviewed PLOS ONE’s open data policy and have revised the Data Availability Statement to clearly describe our data sharing plan. The dataset will be made freely accessible upon acceptance, with all potentially identifiable information removed to ensure compliance with ethical approvals and data protection requirements. All data are freely available in the Zenodo (**[**https://doi.org/10.5281/zenodo.17149348**](https://doi.org/10.5281/zenodo.17149348)**).**

**Comment 3.** If the reviewer comments include a recommendation to cite specific previously published works, please review and evaluate these publications to determine whether they are relevant and should be cited. There is no requirement to cite these works unless the editor has indicated otherwise.

**Response:** We thank the editor for this clarification. We have carefully reviewed the publications recommended by the reviewers and assessed their relevance to our study. Where appropriate, relevant works have been incorporated into the manuscript. Citations that were not directly applicable to our study objectives or context were not included.

**Comment 4.** Additional Editor Comments:

The manuscript has merit and could be considered for publication; however, the authors need to thoroughly address all comments raised by Reviewer 2 before it can move forward.

**Response: We appreciate the editor’s assessment of the manuscript. All comments raised by Reviewer 2 have now been thoroughly addressed. The manuscript has been substantially revised to improve clarity, methodological rigor, citation accuracy, and interpretation of findings.**

**A detailed, point-by-point response to each reviewer comment has been provided, and all corresponding changes have been clearly indicated in the revised manuscript.**

**Reviewer #1:**

**Comment 1.** The introduction should include the burden of diabetic foot ulcer (DFU) in Indonesia.

**Response: Thank you for this important comment. We have revised the Introduction to explicitly describe the burden of diabetes and DFU in Indonesia, including national diabetes prevalence, the clinical and health-system impact of DFU, and the lack of country-specific survival data following amputation.**

**Comment 2.** The term “diabetics” should be avoided. Instead, use “people with diabetes”.

**Response: We appreciate this suggestion and fully agree. The term “diabetics” has been replaced throughout the manuscript.**

**Comment 3.** In the Introduction section, the first 2-3 sentences do not add value. The content is disorganized. The introduction should be rewritten to:

define the disease burden of DFU (global and country data),

Add morbidity and mortality statistics,

explain the significance of the problem and existing gaps in knowledge or practice, conclude with clearly stated study objectives that would address the existing gaps.

**Response: Thank you for this constructive feedback. We have completely rewritten the Introduction to improve clarity, structure, and scientific coherence.**

**Comment 4.** Methods section: Avoid repetition, particularly in describing the study design and study area.

**Response: We thank the reviewer for this comment. The Methods section has been revised to eliminate redundancy between the study design and study area descriptions. The study design has been streamlined, and contextual details regarding the hospital setting are now presented exclusively in the study area subsection.**

**Comment 5.** The exclusion criteria are not sufficiently defined. It is unclear whether patients with a history of amputation or recent amputation prior to hospital presentation were included or excluded.

**Response: We appreciate this important observation. The exclusion criteria have been clarified to explicitly state whether patients with prior amputations or amputations occurring before hospital presentation were included or excluded. Patients who underwent amputation prior to their first DFU-related presentation were excluded, while those with a history of amputation unrelated to the index DFU episode were included.**

**Comment 6.** Clarification is needed regarding patient inclusion criteria. Both patients presenting with new ulcers and those with non-healing ulcers referred to a tertiary center were included. However, the authors have considered the time of presentation to the hospital as the time of diagnosis. This needs to be adequately explained.

**Response: Thank you for this comment. We have clarified that both newly diagnosed and chronic or non-healing DFU cases referred to the tertiary center were included. The date of first DFU-related presentation to the hospital was defined as the date of diagnosis, as this represents the first standardized clinical documentation available in this retrospective cohort. This rationale has now been explicitly stated in the Methods section.**

**Comment 7.** BMI has been given due importance in methods and results but was there any association of BMI with amputation has been reported in previously published studies? Please clarify. Reference cited does not corroborate with the statements.

**Response: Thank you for this important comment. We have clarified the rationale for including BMI as an exposure variable in the Methods section by highlighting its established association with nutritional status, wound healing, and amputation risk in patients with diabetic foot ulcers. We have revised the Discussion to clarify the rationale for BMI-related findings and to avoid overinterpretation of the obesity paradox in the context of DFU amputation. The revised text emphasizes the role of undernutrition, frailty, and impaired wound healing rather than a protective effect of obesity, and references have been adjusted to ensure appropriate support for each statement.**

**Comment 8.** There are multiple factors that could impact the survival and amputation outcomes in patients with diabetic foot ulcers like duration of diabetes, glycemic status, smoking & alcohol use, SES, infection status, Low albumin & anemia should be evaluated as far as available in the medical records.

**Response: We appreciate this valuable suggestion. We agree that factors such as duration of diabetes, glycemic control, smoking, alcohol use, socioeconomic status, infection status, and nutritional biomarkers are important determinants of DFU outcomes. However, these variables were not consistently documented in the retrospective electronic medical records and therefore could not be included in the analysis. This has now been explicitly stated in the Methods and acknowledged as a limitation of the study.**

**Comment 9.** References at multiple places are not appropriately cited.

**Response: Thank you for highlighting this issue. We have carefully reviewed all citations throughout the manuscript and corrected inappropriate or indirect references. Citations have been updated to ensure that each statement is supported by relevant and appropriate literature, particularly in the Introduction and Discussion sections. General statements are now supported by review articles, and outcome-specific claims are cited using DFU-focused studies.**

**Reviewer #2:**

Dear authors, This is a well-written manuscript. The study has merit, and I particularly appreciate the discussion section, where each contributing factor is clearly and thoughtfully addressed.

**Comment 1.** I have one question that I suggest you clarify in the Methods section. You reorganized the established six-tier Wagner classification system for ulcers into a modified 3-tiered system. Could you elaborate on the rationale behind this modification?

I have no further comments.

**Response: Thank you for this important question. The six-tier Wagner classification was regrouped into two clinically meaningful categories such as Grades 0-3 (no gangrene) and Grades 4-5 (gangrene) to improve statistical stability and interpretability in the survival analysis. Several original Wagner grades had relatively small sample sizes, which could compromise the reliability of hazard estimates. Grouping the grades based on the presence or absence of gangrene preserved clinical relevance while ensuring sufficient numbers of events within each category for robust modeling. The rationale for this modification has now been explicitly clarified in the Methods section. For the medical reason, we focus for gangrene group because gangrene is a critical risk factor for amputation in diabetic foot ulcers because it represents irreversible tissue death (necrosis), often combined with severe, rapidly spreading infection and/or lack of blood supply, necessitating surgical removal of the affected part to save the rest of the limb or even the patient’s life.**
