# Supplementary material for: Survival analysis of time to amputation following diabetic foot ulcer diagnosis among Indonesians
Source: PLoS One. 2026 Jun 16;21(6):e0343399. doi: 10.1371/journal.pone.0343399 (PMC13271469; doi:10.1371/journal.pone.0343399)
Supplement: Peer review history

# Author comment: Author response to Decision Letter 0

**DOI:** 10.1371/journal.pone.0343399.r001

4 Oct 2025

Attachment Submitted filename: Response to editor comments.docx

# Peer review: Decision Letter 0

**DOI:** 10.1371/journal.pone.0343399.r002

**Authors:** Shailesh Shahi

11 Dec 2025

PONE-D-25-51099

Survival Analysis of Time to Amputation following Diabetic Foot Ulcer Diagnosis among Indonesians

PLOS One

Dear Dr. Laohasiriwong,

Thank you for submitting your manuscript to PLOS ONE. After careful consideration, we feel that it has merit but does not fully meet PLOS ONE’s publication criteria as it currently stands. Therefore, we invite you to submit a revised version of the manuscript that addresses the points raised during the review process.

Please submit your revised manuscript by Jan 25 2026 11:59PM. If you will need more time than this to complete your revisions, please reply to this message or contact the journal office at plosone@plos.org. When you're ready to submit your revision, log on to https://www.editorialmanager.com/pone/ and select the 'Submissions Needing Revision' folder to locate your manuscript file.

Please include the following items when submitting your revised manuscript:

- A rebuttal letter that responds to each point raised by the academic editor and reviewer(s). You should upload this letter as a separate file labeled 'Response to Reviewers'.
- A marked-up copy of your manuscript that highlights changes made to the original version. You should upload this as a separate file labeled 'Revised Manuscript with Track Changes'.
- An unmarked version of your revised paper without tracked changes. You should upload this as a separate file labeled 'Manuscript'.

If you would like to make changes to your financial disclosure, please include your updated statement in your cover letter. Guidelines for resubmitting your figure files are available below the reviewer comments at the end of this letter.

If applicable, we recommend that you deposit your laboratory protocols in protocols.io to enhance the reproducibility of your results. Protocols.io assigns your protocol its own identifier (DOI) so that it can be cited independently in the future. For instructions see: https://journals.plos.org/plosone/s/submission-guidelines#loc-laboratory-protocols. Additionally, PLOS ONE offers an option for publishing peer-reviewed Lab Protocol articles, which describe protocols hosted on protocols.io. Read more information on sharing protocols at https://plos.org/protocols?utm_medium=editorial-email&utm_source=authorletters&utm_campaign=protocols.

We look forward to receiving your revised manuscript.

Kind regards,

Shailesh Kumar Shahi, PhD

Academic Editor

PLOS One

Journal Requirements:

When submitting your revision, we need you to address these additional requirements.

1.Please ensure that your manuscript meets PLOS ONE's style requirements, including those for file naming. The PLOS ONE style templates can be found at

https://journals.plos.org/plosone/s/file?id=wjVg/PLOSOne_formatting_sample_main_body.pdf and

https://journals.plos.org/plosone/s/file?id=ba62/PLOSOne_formatting_sample_title_authors_affiliations.pdf

2. When completing the data availability statement of the submission form, you indicated that you will make your data available on acceptance. We strongly recommend all authors decide on a data sharing plan before acceptance, as the process can be lengthy and hold up publication timelines. Please note that, though access restrictions are acceptable now, your entire data will need to be made freely accessible if your manuscript is accepted for publication. This policy applies to all data except where public deposition would breach compliance with the protocol approved by your research ethics board. If you are unable to adhere to our open data policy, please kindly revise your statement to explain your reasoning and we will seek the editor's input on an exemption. Please be assured that, once you have provided your new statement, the assessment of your exemption will not hold up the peer review process.

3. If the reviewer comments include a recommendation to cite specific previously published works, please review and evaluate these publications to determine whether they are relevant and should be cited. There is no requirement to cite these works unless the editor has indicated otherwise.

Additional Editor Comments:

The manuscript has merit and could be considered for publication; however, the authors need to thoroughly address all comments raised by Reviewer 2 before it can move forward.

[Note: HTML markup is below. Please do not edit.]

Reviewers' comments:

Reviewer's Responses to Questions

**Comments to the Author**

1. Is the manuscript technically sound, and do the data support the conclusions?

The manuscript must describe a technically sound piece of scientific research with data that supports the conclusions. Experiments must have been conducted rigorously, with appropriate controls, replication, and sample sizes. The conclusions must be drawn appropriately based on the data presented.

Reviewer #1: Partly

Reviewer #2: Yes

**********

2. Has the statistical analysis been performed appropriately and rigorously?

Reviewer #1: I Don't Know

Reviewer #2: Yes

**********

3. Have the authors made all data underlying the findings in their manuscript fully available?

The PLOS Data policy requires authors to make all data underlying the findings described in their manuscript fully available without restriction, with rare exception (please refer to the Data Availability Statement in the manuscript PDF file). The data should be provided as part of the manuscript or its supporting information, or deposited to a public repository. For example, in addition to summary statistics, the data points behind means, medians and variance measures should be available. If there are restrictions on publicly sharing data—e.g. participant privacy or use of data from a third party—those must be specified.

Reviewer #1: Yes

Reviewer #2: Yes

**********

4. Is the manuscript presented in an intelligible fashion and written in standard English?

PLOS ONE does not copyedit accepted manuscripts, so the language in submitted articles must be clear, correct, and unambiguous. Any typographical or grammatical errors should be corrected at revision, so please note any specific errors here.

Reviewer #1: No

Reviewer #2: Yes

**********

5. Review Comments to the Author

Please use the space provided to explain your answers to the questions above. You may also include additional comments for the author, including concerns about dual publication, research ethics, or publication ethics. (Please upload your review as an attachment if it exceeds 20,000 characters)

Reviewer #1: 1. The introduction should include the burden of diabetic foot ulcer (DFU) in Indonesia.

2. The term “diabetics” should be avoided. Instead, use “people with diabetes”.

3. In the Introduction section, the first 2–3 sentences do not add value. The content is disorganized. The introduction should be rewritten to:

• define the disease burden of DFU (global and country data),

• Add morbidity and mortality statistics,

• explain the significance of the problem and existing gaps in knowledge or practice,

• conclude with clearly stated study objectives that would address the existing gaps.

4. Methods section: Avoid repetition, particularly in describing the study design and study area.

5. The exclusion criteria are not sufficiently defined. It is unclear whether patients with a history of amputation or recent amputation prior to hospital presentation were included or excluded.

6. Clarification is needed regarding patient inclusion criteria. Both patients presenting with new ulcers and those with non-healing ulcers referred to a tertiary center were included. However, the authors have considered the time of presentation to the hospital as the time of diagnosis. This needs to be adequately explained.

7. BMI has been given due importance in methods and results but was there any association of BMI with amputation has been reported in previously published studies? Please clarify. Reference cited does not corroborate with the statements.

8. There are multiple factors that could impact the survival and amputation outcomes in patients with diabetic foot ulcers like duration of diabetes, glycemic status, smoking & alcohol use, SES, infection status, Low albumin & anemia should be evaluated as far as available in the medical records.

9. References at multiple places are not appropriately cited.

Reviewer #2: Dear authors,

This is a well-written manuscript. The study has merit, and I particularly appreciate the discussion section, where each contributing factor is clearly and thoughtfully addressed.

I have one question that I suggest you clarify in the Methods section. You reorganized the established six-tier Wagner classification system for ulcers into a modified 3-tiered system.

Could you elaborate on the rationale behind this modification?

I have no further comments.

**********

6. PLOS authors have the option to publish the peer review history of their article (what does this mean?). If published, this will include your full peer review and any attached files.

If you choose “no”, your identity will remain anonymous but your review may still be made public.

**Do you want your identity to be public for this peer review?**  For information about this choice, including consent withdrawal, please see our Privacy Policy.

Reviewer #1: No

Reviewer #2: **No**

**********

[NOTE: If reviewer comments were submitted as an attachment file, they will be attached to this email and accessible via the submission site. Please log into your account, locate the manuscript record, and check for the action link "View Attachments". If this link does not appear, there are no attachment files.]

To ensure your figures meet our technical requirements, please review our figure guidelines: https://journals.plos.org/plosone/s/figures

You may also use PLOS’s free figure tool, NAAS, to help you prepare publication quality figures: https://journals.plos.org/plosone/s/figures#loc-tools-for-figure-preparation.

NAAS will assess whether your figures meet our technical requirements by comparing each figure against our figure specifications.

# Author comment: Author response to Decision Letter 1

**DOI:** 10.1371/journal.pone.0343399.r003

18 Dec 2025

Response to Reviewer

Journal Name: PLOS One

Manuscript ID: PONE-D-25-51099

Title: Survival Analysis of Time to Amputation following Diabetic Foot Ulcer Diagnosis among Indonesians

Authors: Lianawati1 , Wongsa Laohasiriwong2* , Kyaw Min Htike1 , Kavin Thinkhamrop2 , Roshan Kumar Mahato2

Dear Editor,

Thank you very much for your efforts on our manuscript. We are thankful for the editor and reviewer’s critical comments and have revised the manuscript accordingly. The responses to editor and reviewer comments are summarized in this letter point-by-point.

We appreciate your precious time in reviewing our manuscript, and we would be grateful if you would consider this manuscript for publication after review.

Yours sincerely,

Wongsa Laohasiriwong

Corresponding Author

Faculty of Public Health, Khon Kaen University, Thailand

Response to Editor

Comment 1. Please ensure that your manuscript meets PLOS ONE's style requirements, including those for file naming. The PLOS ONE style templates can be found at https://journals.plos.org/plosone/ s/file?id=wjVg/PLOSOne_formatting_sample_ main_body .pdf and https://journals.plos. org /plosone/s/file?id=ba62/PLOSOne_formatting _sample_ title_authors_affiliations.pdf

Response: Thank you for this guidance. We have revised the manuscript to fully comply with PLOS ONE formatting and style requirements, including file naming, structure, headings, and layout, using the official PLOS ONE formatting templates for the main body and title/authors/affiliations. The revised files have been uploaded accordingly.

Comment 2. When completing the data availability statement of the submission form, you indicated that you will make your data available on acceptance. We strongly recommend all authors decide on a data sharing plan before acceptance, as the process can be lengthy and hold up publication timelines. Please note that, though access restrictions are acceptable now, your entire data will need to be made freely accessible if your manuscript is accepted for publication. This policy applies to all data except where public deposition would breach compliance with the protocol approved by your research ethics board. If you are unable to adhere to our open data policy, please kindly revise your statement to explain your reasoning and we will seek the editor's input on an exemption. Please be assured that, once you have provided your new statement, the assessment of your exemption will not hold up the peer review process.

Response: Thank you for this important clarification. We have carefully reviewed PLOS ONE’s open data policy and have revised the Data Availability Statement to clearly describe our data sharing plan. The dataset will be made freely accessible upon acceptance, with all potentially identifiable information removed to ensure compliance with ethical approvals and data protection requirements. All data are freely available in the Zenodo (https://doi.org/10.5281/zenodo.17149348).

Comment 3. If the reviewer comments include a recommendation to cite specific previously published works, please review and evaluate these publications to determine whether they are relevant and should be cited. There is no requirement to cite these works unless the editor has indicated otherwise.

Response: We thank the editor for this clarification. We have carefully reviewed the publications recommended by the reviewers and assessed their relevance to our study. Where appropriate, relevant works have been incorporated into the manuscript. Citations that were not directly applicable to our study objectives or context were not included.

Comment 4. Additional Editor Comments:

The manuscript has merit and could be considered for publication; however, the authors need to thoroughly address all comments raised by Reviewer 2 before it can move forward.

Response: We appreciate the editor’s assessment of the manuscript. All comments raised by Reviewer 2 have now been thoroughly addressed. The manuscript has been substantially revised to improve clarity, methodological rigor, citation accuracy, and interpretation of findings.

A detailed, point-by-point response to each reviewer comment has been provided, and all corresponding changes have been clearly indicated in the revised manuscript.

Reviewer #1:

Comment 1. The introduction should include the burden of diabetic foot ulcer (DFU) in Indonesia.

Response: Thank you for this important comment. We have revised the Introduction to explicitly describe the burden of diabetes and DFU in Indonesia, including national diabetes prevalence, the clinical and health-system impact of DFU, and the lack of country-specific survival data following amputation.

Comment 2. The term “diabetics” should be avoided. Instead, use “people with diabetes”.

Response: We appreciate this suggestion and fully agree. The term “diabetics” has been replaced throughout the manuscript.

Comment 3. In the Introduction section, the first 2-3 sentences do not add value. The content is disorganized. The introduction should be rewritten to:

define the disease burden of DFU (global and country data),

Add morbidity and mortality statistics,

explain the significance of the problem and existing gaps in knowledge or practice, conclude with clearly stated study objectives that would address the existing gaps.

Response: Thank you for this constructive feedback. We have completely rewritten the Introduction to improve clarity, structure, and scientific coherence.

Comment 4. Methods section: Avoid repetition, particularly in describing the study design and study area.

Response: We thank the reviewer for this comment. The Methods section has been revised to eliminate redundancy between the study design and study area descriptions. The study design has been streamlined, and contextual details regarding the hospital setting are now presented exclusively in the study area subsection.

Comment 5. The exclusion criteria are not sufficiently defined. It is unclear whether patients with a history of amputation or recent amputation prior to hospital presentation were included or excluded.

Response: We appreciate this important observation. The exclusion criteria have been clarified to explicitly state whether patients with prior amputations or amputations occurring before hospital presentation were included or excluded. Patients who underwent amputation prior to their first DFU-related presentation were excluded, while those with a history of amputation unrelated to the index DFU episode were included.

Comment 6. Clarification is needed regarding patient inclusion criteria. Both patients presenting with new ulcers and those with non-healing ulcers referred to a tertiary center were included. However, the authors have considered the time of presentation to the hospital as the time of diagnosis. This needs to be adequately explained.

Response: Thank you for this comment. We have clarified that both newly diagnosed and chronic or non-healing DFU cases referred to the tertiary center were included. The date of first DFU-related presentation to the hospital was defined as the date of diagnosis, as this represents the first standardized clinical documentation available in this retrospective cohort. This rationale has now been explicitly stated in the Methods section.

Comment 7. BMI has been given due importance in methods and results but was there any association of BMI with amputation has been reported in previously published studies? Please clarify. Reference cited does not corroborate with the statements.

Response: Thank you for this important comment. We have clarified the rationale for including BMI as an exposure variable in the Methods section by highlighting its established association with nutritional status, wound healing, and amputation risk in patients with diabetic foot ulcers. We have revised the Discussion to clarify the rationale for BMI-related findings and to avoid overinterpretation of the obesity paradox in the context of DFU amputation. The revised text emphasizes the role of undernutrition, frailty, and impaired wound healing rather than a protective effect of obesity, and references have been adjusted to ensure appropriate support for each statement.

Comment 8. There are multiple factors that could impact the survival and amputation outcomes in patients with diabetic foot ulcers like duration of diabetes, glycemic status, smoking & alcohol use, SES, infection status, Low albumin & anemia should be evaluated as far as available in the medical records.

Response: We appreciate this valuable suggestion. We agree that factors such as duration of diabetes, glycemic control, smoking, alcohol use, socioeconomic status, infection status, and nutritional biomarkers are important determinants of DFU outcomes. However, these variables were not consistently documented in the retrospective electronic medical records and therefore could not be included in the analysis. This has now been explicitly stated in the Methods and acknowledged as a limitation of the study.

Comment 9. References at multiple places are not appropriately cited.

Response: Thank you for highlighting this issue. We have carefully reviewed all citations throughout the manuscript and corrected inappropriate or indirect references. Citations have been updated to ensure that each statement is supported by relevant and appropriate literature, particularly in the Introduction and Discussion sections. General statements are now supported by review articles, and outcome-specific claims are cited using DFU-focused studies.

Reviewer #2:

Dear authors, This is a well-written manuscript. The study has merit, and I particularly appreciate the discussion section, where each contributing factor is clearly and thoughtfully addressed.

Comment 1. I have one question that I suggest you clarify in the Methods section. You reorganized the established six-tier Wagner classification system for ulcers into a modified 3-tiered system. Could you elaborate on the rationale behind this modification?

I have no further comments.

Response: Thank you for this important question. The six-tier Wagner classification was regrouped into two clinically meaningful categories such as Grades 0-3 (no gangrene) and Grades 4-5 (gangrene) to improve statistical stability and interpretability in the survival analysis. Several original Wagner grades had relatively small sample sizes, which could compromise the reliability of hazard estimates. Grouping the grades based on the presence or absence of gangrene preserved clinical relevance while ensuring sufficient numbers of events within each category for robust modeling. The rationale for this modification has now been explicitly clarified in the Methods section. For the medical reason, we focus for gangrene group because gangrene is a critical risk factor for amputation in diabetic foot ulcers because it represents irreversible tissue death (necrosis), often combined with severe, rapidly spreading infection and/or lack of blood supply, necessitating surgical removal of the affected part to save the rest of the limb or even the patient’s life.

Attachment Submitted filename: Response to reviewer.docx

# Peer review: Decision Letter 1

**DOI:** 10.1371/journal.pone.0343399.r004

**Authors:** Shailesh Shahi

6 Feb 2026

Survival Analysis of Time to Amputation following Diabetic Foot Ulcer Diagnosis among Indonesians

PONE-D-25-51099R1

Dear Dr. Laohasiriwong,

We’re pleased to inform you that your manuscript has been judged scientifically suitable for publication and will be formally accepted for publication once it meets all outstanding technical requirements.

Within one week, you’ll receive an e-mail detailing the required amendments. When these have been addressed, you’ll receive a formal acceptance letter and your manuscript will be scheduled for publication.

An invoice will be generated when your article is formally accepted. Please note, if your institution has a publishing partnership with PLOS and your article meets the relevant criteria, all or part of your publication costs will be covered. Please make sure your user information is up-to-date by logging into Editorial Manager at Editorial Manager® and clicking the ‘Update My Information' link at the top of the page. For questions related to billing, please contact billing support.

If your institution or institutions have a press office, please notify them about your upcoming paper to help maximize its impact. If they’ll be preparing press materials, please inform our press team as soon as possible -- no later than 48 hours after receiving the formal acceptance. Your manuscript will remain under strict press embargo until 2 pm Eastern Time on the date of publication. For more information, please contact onepress@plos.org.

Kind regards,

Shailesh Kumar Shahi, PhD

Academic Editor

PLOS One

Additional Editor Comments (optional):

It is highly recommended that all cited references accurately match the corresponding statements in the text. I have reviewed the manuscript and noticed that, in several instances, the references cited do not appropriately support the associated claims. Kindly cross-check all references carefully and correct them during the proofreading and production stages of the manuscript.

Reviewers' comments:

Reviewer's Responses to Questions

**Comments to the Author**

1. If the authors have adequately addressed your comments raised in a previous round of review and you feel that this manuscript is now acceptable for publication, you may indicate that here to bypass the “Comments to the Author” section, enter your conflict of interest statement in the “Confidential to Editor” section, and submit your "Accept" recommendation.

Reviewer #1: All comments have been addressed

**********

2. Is the manuscript technically sound, and do the data support the conclusions?

The manuscript must describe a technically sound piece of scientific research with data that supports the conclusions. Experiments must have been conducted rigorously, with appropriate controls, replication, and sample sizes. The conclusions must be drawn appropriately based on the data presented.

Reviewer #1: Yes

**********

3. Has the statistical analysis been performed appropriately and rigorously?

Reviewer #1: I Don't Know

**********

4. Have the authors made all data underlying the findings in their manuscript fully available?

The PLOS Data policy requires authors to make all data underlying the findings described in their manuscript fully available without restriction, with rare exception (please refer to the Data Availability Statement in the manuscript PDF file). The data should be provided as part of the manuscript or its supporting information, or deposited to a public repository. For example, in addition to summary statistics, the data points behind means, medians and variance measures should be available. If there are restrictions on publicly sharing data—e.g. participant privacy or use of data from a third party—those must be specified.

Reviewer #1: Yes

**********

5. Is the manuscript presented in an intelligible fashion and written in standard English?

PLOS ONE does not copyedit accepted manuscripts, so the language in submitted articles must be clear, correct, and unambiguous. Any typographical or grammatical errors should be corrected at revision, so please note any specific errors here.

Reviewer #1: (No Response)

**********

6. Review Comments to the Author

Please use the space provided to explain your answers to the questions above. You may also include additional comments for the author, including concerns about dual publication, research ethics, or publication ethics. (Please upload your review as an attachment if it exceeds 20,000 characters)

Reviewer #1: No further comments to authors. Most of comments have been addressed. However, it seems that at certain places cited references does not match with the quoted texts.

**********

7. PLOS authors have the option to publish the peer review history of their article (what does this mean?). If published, this will include your full peer review and any attached files.

If you choose “no”, your identity will remain anonymous but your review may still be made public.

**Do you want your identity to be public for this peer review?**  For information about this choice, including consent withdrawal, please see our Privacy Policy.

Reviewer #1: No

**********

# Editor report: Acceptance letter

**DOI:** 10.1371/journal.pone.0343399.r005

**Authors:** Shailesh Shahi

PONE-D-25-51099R1

PLOS One

Dear Dr. Laohasiriwong,

I'm pleased to inform you that your manuscript has been deemed suitable for publication in PLOS One. Congratulations! Your manuscript is now being handed over to our production team.

At this stage, our production department will prepare your paper for publication. This includes ensuring the following:

* All references, tables, and figures are properly cited

* All relevant supporting information is included in the manuscript submission,

* There are no issues that prevent the paper from being properly typeset

You will receive further instructions from the production team, including instructions on how to review your proof when it is ready. Please keep in mind that we are working through a large volume of accepted articles, so please give us a few days to review your paper and let you know the next and final steps.

Lastly, if your institution or institutions have a press office, please let them know about your upcoming paper now to help maximize its impact. If they'll be preparing press materials, please inform our press team within the next 48 hours. Your manuscript will remain under strict press embargo until 2 pm Eastern Time on the date of publication. For more information, please contact onepress@plos.org.

You will receive an invoice from PLOS for your publication fee after your manuscript has reached the completed accept phase. If you receive an email requesting payment before acceptance or for any other service, this may be a phishing scheme. Learn how to identify phishing emails and protect your accounts at https://explore.plos.org/phishing.

If we can help with anything else, please email us at customercare@plos.org.

Thank you for submitting your work to PLOS ONE and supporting open access.

Kind regards,

PLOS ONE Editorial Office Staff

on behalf of

Dr. Shailesh Kumar Shahi

Academic Editor

PLOS One
